# Photothermally enhanced antibacterial wound healing using albumin-loaded tanshinone IIA and IR780 nanoparticles

**DOI:** 10.3389/fbioe.2024.1487660

**Published:** 2024-10-23

**Authors:** Haidong Chen, Yimei Li, Dexuan Chen, Yong Fang, Xuchu Gong, Kaikai Wang, Chaoqun Ma

**Affiliations:** ^1^ Department of General Surgery, Nantong Hospital Affiliated to Nanjing University of Chinese Medicine, Nantong, China; ^2^ Department of General Surgery, Jiangsu Province Hospital of Chinese Medicine, Affiliated Hospital of Nanjing University of Chinese Medicine, Nanjing, China; ^3^ School of Pharmacy, Nantong University, Nantong, China

**Keywords:** tanshinone IIA, albumin nanoparticles, wound healing, photothermal therapy, antibacterial

## Abstract

Chronic and infected wounds, particularly those caused by bacterial infections, present significant challenges in medical treatment. This study aimed to develop a novel nanoparticle formulation to enhance wound healing by combining antimicrobial and photothermal therapy using albumin as a carrier for Tanshinone IIA and the near-infrared photothermal agent IR780. The nanoparticles were synthesized to exploit the antimicrobial effects of Tanshinone IIA and the photothermal properties of IR780 when exposed to near-infrared laser irradiation. Characterization of the nanoparticles was performed using Transmission Electron Microscopy (TEM) and spectroscopic analysis to confirm their successful synthesis. *In vitro* antibacterial activity was evaluated using cultures of methicillin-resistant *Staphylococcus aureus* (MRSA), and *in vivo* efficacy was tested in a mouse model of MRSA-infected wounds. Wound healing progression was assessed over 16 days, with statistical analysis performed using two-way ANOVA followed by Tukey’s post-hoc test. The nanoparticles demonstrated significant photothermal properties, enhancing bacterial eradication and promoting the controlled release of Tanshinone IIA. *In vitro* studies showed superior antibacterial activity, especially under photothermal activation, leading to a substantial reduction in bacterial viability in MRSA cultures. In vivo, nanoparticle treatment combined with near-infrared laser irradiation significantly improved wound closure rates compared to controls and treatments without photothermal activation. By the 16th day post-treatment, significant improvements in wound healing were observed, highlighting the potential of the combined photothermal and pharmacological approach. These findings suggest that albumin-loaded nanoparticles containing Tanshinone IIA and IR780, activated by near-infrared light, could offer an effective therapeutic strategy for managing chronic and infected wounds, promoting both infection control and tissue repair.

## 1 Introduction

Skin is the largest organ in the human body. It not only protects the body from external microbes, chemicals, and physical stimuli, but also plays critical roles in regulating body temperature, maintaining moisture balance, and sensing the external environment (Nestle et al., 2009; Proksch et al., 2008; Wang et al., 2024). However, skin injuries can be caused by various factors such as trauma, burns, and surgery. These injuries disrupt the integrity of the skin, leading to imbalances in internal and external environments and potential risks of infection (Zhou et al., 2023). Once the skin is injured, the body immediately initiates a series of complex biological responses to address the damage and promote the healing and repair process. This process involves the coordinated action of multiple cell types, signaling molecules, and biological processes (Mahdavian Delavary et al., 2011; Sorg et al., 2017). Initially, the inflammatory response phase triggers vasodilation, increased vascular permeability, and the infiltration of inflammatory cells, aiming to clear dead tissue, pathogens, and cellular debris (Xiao et al., 2023). Subsequently, the new tissue formation stage involves the migration and proliferation of fibroblasts and the synthesis of collagen, thus filling the wound and forming preliminary repair tissue. Finally, the tissue remodeling stage involves further remodeling and regeneration of the repair tissue, as well as the regeneration of blood vessels and nerves, to achieve complete wound healing and functional restoration (Takeo et al., 2015).

Managing skin injuries is not just about simple wound treatment; it requires a comprehensive consideration of the biological processes of wound healing and taking appropriate measures to promote the healing process. This might include cleaning the wound, local antibacterial treatment, appropriate dressing selection, nutritional support, and surgical intervention when necessary (Sun et al., 2014). Effective management can speed up wound healing and reduce the occurrence of infections and complications. After skin damage, its ability to resist bacteria significantly decreases; exposure to a moist environment provides suitable conditions for bacterial growth, potentially leading to infectious wounds. A large number of bacteria in an infectious wound will recruit inflammatory cells, such as macrophages and neutrophils, to the injury site (Eming et al., 2007; Naik et al., 2017). In the early stages of skin repair, macrophages tend to frequently differentiate into M1-type macrophages, leading to the appearance of acute inflammation. Acute inflammation is the body’s protective response to the injured site (Shi et al., 2022). In the microenvironment, inflammatory cells control wound infections by phagocytosing bacteria, thereby promoting wound healing. However, persistent bacterial infections can lead to prolonged chronic inflammation and delay the transition of the wound healing process to the next proliferation stage, further delaying normal wound healing and turning it into a chronic wound (Chiller et al., 2001; Zegadlo et al., 2023).

Traditional wound dressings (gauze, bandages, etc.) only have simple functions such as quick hemostasis and absorption of exudates. Their singular functionality during the wound healing process can lead to secondary wound infections and the recurrence of inflammation (Cullen and Gefen, 2023). Therefore, there is a need to develop a multifunctional nanomedicine to comprehensively treat the problem of chronic wounds that are difficult to heal (Wolf et al., 2009). This type of multifunctional nanomedicine needs to have strong antibacterial capabilities, anti-inflammatory properties, and the ability to regulate the local immune environment.

Photothermal therapy (PTT) has attracted attention due to its novel mechanism, which differs from traditional therapies (Su et al., 2019; Duan et al., 2023). Traditional antibiotics, such as penicillin and its related beta-lactams, are the most widely used antibiotics that kill bacteria by inhibiting bacterial cell wall synthesis through binding to penicillin-binding proteins (Chen et al., 2020). In contrast, PTT directly targets photothermal agents such as gold, molybdenum disulfide, near-infrared organic small molecules, and graphene nanomaterials, which have high photothermal conversion efficiency under near-infrared laser (Yu et al., 2019). Nanomaterials under near-infrared convert light energy into heat energy, causing irreversible damage to bacteria. Compared to traditional therapies, PTT is an ideal antimicrobial treatment method because it causes less trauma, has a shorter treatment duration, strong penetration ability, and fewer side effects (Chen et al., 2020).

Salvia miltiorrhiza, also known as Danshen, is a traditional Chinese herb made from the dried roots and rhizomes of the plant (Jiang et al., 2019). It has been found to alleviate pain, promote blood circulation, and eliminate blood stasis. Modern pharmacological research has shown that Danshen can dilate coronary arteries, prevent myocardial ischemia and myocardial infarction, improve microcirculation, and reduce myocardial oxygen consumption (Lin and Hsieh, 2010). Numerous experimental and clinical studies have reported benefits to the heart during pathological processes such as myocardial ischemia, myocardial infarction, and reperfusion injury (Zhou et al., 2005). Tanshinone IIA is a representative lipid-soluble component of Danshen, and other tanshinones and the hydrophilic component (Danshen) also play important roles in the pharmacological activities of Danshen in treating various diseases (Zhang et al., 2020; Zhang et al., 2012). Terpenoids are easily reduced to dihydro derivatives, then oxidized and readily transformed. Quinone compounds play a role in transferring electrons in the metabolic products of organisms, exhibiting various biological activities by promoting or interfering with various biochemical reactions in the body. They serve as coenzymes in biological reactions to promote some biochemical processes or disrupt their action, thus displaying various pharmacological effects such as anti-atherosclerosis, anti-myocardial ischemia, anti-arrhythmic, repairing vascular endothelial cells, improving coronary blood flow, anti-myocardial hypertrophy, and anti-tumor effects (Guo et al., 2020). Research has found that Tanshinone IIA plays an important role in the activation, development, and normal function of immune cells. Tanshinone IIA has been shown to exhibit significant antibacterial activity against both Gram-positive and Gram-negative bacteria. Studies have demonstrated that Tanshinone IIA can disrupt bacterial cell membranes, inhibit biofilm formation, and reduce bacterial adhesion (Li and Zhou, 2018; Wang et al., 2023). Tanshinone IIA participates in both innate and acquired immune responses, promoting the various stages of the inflammatory pathway (Gong et al., 2020).

To date, a variety of biomaterials have been used for rapid wound healing, including electrospun nanofibers, porous foams, biocompatible membranes, nanopreparations, and functional hydrogels (Zhang et al., 2023; Naderi et al., 2018). Among these, nanopreparations are the most widely used, especially those using albumin as a carrier (da Silva et al., 2018). Albumin nanoparticles can serve as an effective vehicle for drugs, encapsulating them inside or on the surface to increase drug stability, solubility, and bioavailability (Elzoghby et al., 2012). This type of carrier can achieve controlled drug release by adjusting the size, surface properties, and drug release rates of the nanoparticles, thereby enhancing the therapeutic effects of the drugs.

**FIGURE 1:**
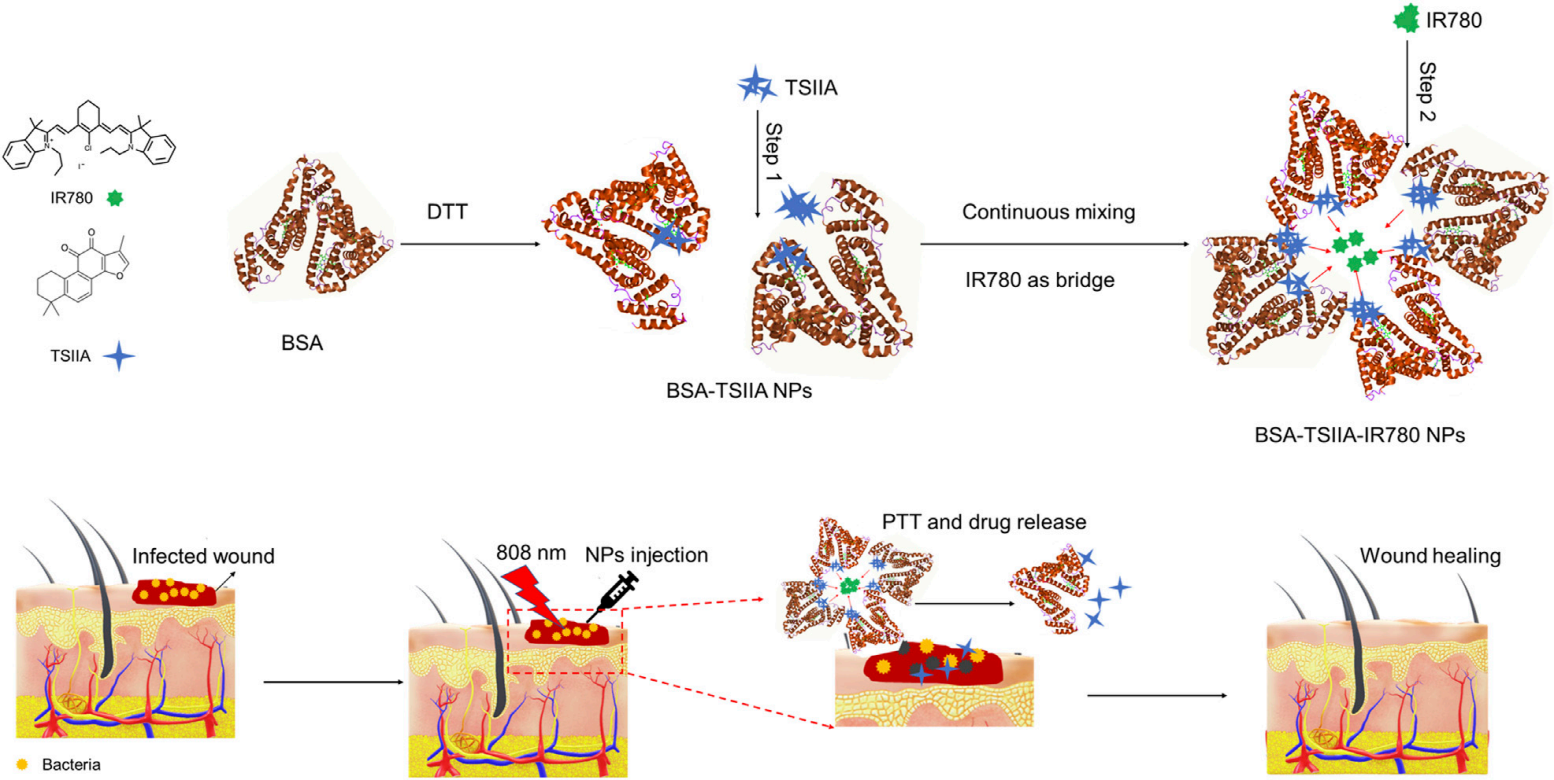
Schematic diagram of the preparation of albumin nanoparticles loaded with Tanshinone IIA and IR780 using the “molecular switch” method and their combined photothermal treatment effect on wound healing caused by bacterial infection. This combination of photothermal therapy and drug release accelerates tissue repair and regeneration, leading to improved wound closure and recovery.

In this study, albumin-loaded Tanshinone IIA and the near-infrared small molecule IR780 nanoparticles were prepared using the “molecular switch” method via a two-step process (Gong et al., 2012). First, the disulfide bonds within the albumin molecule were opened by dithiothreitol (DTT), exposing the hydrophobic regions of albumin. In the second step, the composite nanoparticles were further obtained through the electrostatic adsorption and hydrophobic interactions of IR780 (Figure 1). In the wound healing model, we infected mouse wounds with methicillin-resistant *Staphylococcus aureus* (MRSA) to obtain a clinically relevant model of difficult-to-heal wounds and fully evaluated the combined therapeutic effects of albumin-loaded Tanshinone IIA and IR780 nanoparticles.

## 2 Materials and methods

### 2.1 Materials

IR780 (95%) and Tanshinone IIA (98%) were sourced from Shanghai Macklin Biochemical Co., Ltd., while Bovine Serum Albumin (98%) was acquired from Shanghai Aladdin Reagent Co., Ltd. Dithiothreitol (DTT) was provided by Nanjing Wanqing Chemical Glassware Instrument Co. Ltd. (China). The cell counting kit-8 (CCK-8) was obtained from Dojindo Laboratories (Japan). Unless specified otherwise, all additional reagents were purchased from Nanjing Wanqing Chemical Glassware Instrument Co. Ltd. and were used without further modification.

### 2.2 Preparation and characterization of nanoparticles

Using the “molecular switch” method, Tanshinone IIA (TSIIA) and IR780 (BSA-TSIIA-IR780 NPs) dual-loaded albumin nanoparticles were prepared by a two-step method. Initially, 100 mg of Bovine Serum Albumin (BSA) was completely dissolved in 50 mL of phosphate-buffered saline (PBS). Subsequently, 100 μL of dithiothreitol (DTT) solution (10 mg/mL) was added to the BSA solution at 45°C. During the reaction, DTT effectively opened the hydrophobic spaces within BSA. Then, while continuously stirring, 2 mL of ethanol solution containing Tanshinone IIA (10 mg/mL) was gradually added to the BSA solution, resulting in the formation of BSA-TSIIA nanoparticles (BSA-TSIIA NPs).

In the second step, 1 mL of ethanol solution containing IR780 (2 mg/mL) was added to the aforementioned solution. The interaction of intermolecular electrostatic forces and hydrophobic interactions facilitated the formation of BSA-TSIIA-IR780 nanoparticles. Subsequently, the nanoparticle solution was ultrafiltered three times to remove free IR780, TSIIA, and ethanol. The final concentrated solution was about 3 mL, suitable for subsequent experiments.

The content of IR780 in the BSA-TSIIA-IR780 nanoparticles was analyzed using a spectrophotometric method. Briefly, the BSA-TSIIA-IR780 nanoparticles were degraded by acetonitrile (volume ratio 1:1). Afterwards, the resulting mixture was diluted 25-fold in chloroform and sonicated for 10 min to ensure complete extraction of IR780. The concentration of IR780 was determined by measuring the absorbance at 785 nm using a UV-vis-NIR spectrophotometer (UV-2450, Shimadzu, Japan) and calculating it from the standard curve of IR780 in chloroform. The content of TSIIA was calculated using high-performance liquid chromatography (HPLC) with reference to the standard curve. TSIIA detection was conducted at a wavelength of 270 nm, using a reverse phase C18 column (5 μm, 4.6 mm × 250 mm, Agilent, United States) at 25°C. The flow rate was maintained at 1.0 mL/min. The elution solvents were phase A (methanol) and phase B (water), with a ratio of 85% A to 15% B (Meng et al., 2015). The BSA concentration was determined using the Coomassie Brilliant Blue method.

The particle size of the BSA-TSIIA-IR780 nanoparticles was determined by Dynamic Light Scattering (DLS) using a Zeta Plus (Brookhaven Instruments Corporation, United States). Before and after irradiation with an 808 nm laser (power density of 1w/cm^2^, for 5 min), the nanoparticles were negatively stained with phosphotungstic acid, and their morphology was assessed using a Transmission Electron Microscope (TEM, Hitachi H-600, Japan).

### 2.3 *In Vitro* heating curve

BSA-TSIIA-IR780 nanoparticles were diluted to 0.05 mg/mL (concentration of IR780) and exposed to an 808 nm near-infrared laser at a power density of 1.0 W/cm^2^ for a continuous duration of 3 min. A thermometer was used to measure the temperature every 30 s. BSA-IR780 nanoparticles, free IR780, and PBS were used as controls.

### 2.4 Drug release study

To study the release curve of TSIIA from nanoparticles, 1 mL of BSA-TSIIA-IR780 nanoparticles and BSA-TSIIA nanoparticles were separately placed into dialysis bags (molecular weight cutoff of 3.5 kDa) and immersed in 15 mL of release medium (PBS containing 1% Tween 80, pH 7.4). The release behavior of BSA-TSIIA-IR780 nanoparticles was investigated with and without irradiation by an 808 nm laser (power of 1w/cm^2^ for 5 min). Samples (release solution) were collected at predetermined time points (0–72 h) and the same volume of release medium was replenished. The content of TSIIA was measured by HPLC (as previously described), and the drug release curve was obtained based on the standard curve.

### 2.5 *In Vitro* antibacterial experiment

The model bacterium used in the antibacterial experiment was methicillin-resistant *S. aureus* (MRSA, ATCC 43300). MRSA cells were cultured in lysogeny broth (LB) medium at 37°C under aerobic conditions until they reached mid-exponential growth phase (Liu et al., 2022). To assess the *in vitro* antibacterial activity of various concentrations of BSA-TSIIA-IR780 nanoparticles, a suspension of MRSA (100 μL, 1 × 10^5^ CFU/mL) was added to a 96-well plate, along with a range of concentrations of BSA-TSIIA-IR780 nanoparticles (100 μL; 0.5, 1.0, 2.0, and 5 μg/mL IR780; 5.0, 10.0, 20.0, and 50.0 μg/mL TSIIA), BSA-TSIIA nanoparticles (5.0, 10.0, 20.0, and 50.0 μg/mL TSIIA), and BSA-IR780 nanoparticles (100 μL; 0.5, 1.0, 2.0, and 5 μg/mL IR780). LB solution was used as a control. In the photothermal treatment group, the 96-well plate was irradiated with a near-infrared laser (808 nm, 1.0 W/cm^2^) for 5 min. After incubation for 120 min at 37°C, CCK-8 solution (10 µL/well) was added to the 96-well plate. After incubating for 30 min in the dark at room temperature, the plate was placed in an enzyme-linked immunosorbent assay reader to measure the absorbance at 450 nm, which reflects the number of live bacteria in each well. Each group included five replicate wells. Cell viability was expressed as the mean absorbance ±standard deviation (SD) of the five wells per group. The experiment was repeated three times (Xu et al., 2021).

### 2.6 Establishment of mouse wound model and pharmacological study

All animal experiments were conducted in compliance with the Animal Care and Use Committee of Nantong University. For the wound healing model, this experiment used 5 week-old male ICR mice. Prior to wound creation, the mice were given an intraperitoneal injection of an anesthetic mixture (ketamine/xylazine), and then the hair on their dorsal surface was shaved. A skin wound measuring 1 × 1 cm^2^ was created on the dorsal surface of the mice using a 28-gauge needle. Five minutes after creating the wound, each scratch area was inoculated with 40 µL of a suspension containing 1 × 10^8^ CFU/mL MRSA, dispersed in PBS. Twenty-4 h after the wounds were infected with the MRSA suspension, the mice were randomly assigned to different treatment groups (each group, n = 4) as follows: control group (100 µL PBS), BSA-TSIIA NPs (100 μL, 200 μg/mL TSIIA), BSA-TSIIA-IR780 NPs without laser (100 μL, 200 μg/mL TSIIA and 20 μg/mL IR780), BSA-IR780 NPs (100 μL, 20 μg/mL IR780) plus laser, and BSA-TSIIA-IR780 NPs (100 μL, 200 μg/mL TSIIA and 20 μg/mL IR780) plus laser. A near-infrared laser (808 nm, 1.0 W/cm^2^) was used to irradiate the treatment area for 5 min, 24 h after nanoparticle application to the mice. Wound size was measured using a digital caliper and photographed on days 0, 2, 4, 8, and 16. Wound healing rate was calculated using the following equation:

$$\text{Relative wound area }\left(\%\right)=W_{△}\;/\;W_{0}\times 100\%$$

Where W_△_ is the wound area on a specific day, and W_0_ is the wound area on day 0.

### 2.7 Statistical analysis

Comparison between two groups was performed using Student’s t-test. Comparisons among more than two groups were conducted using two-way Analysis of Variance (ANOVA), followed by Tukey post-hoc analysis to compare the means of two groups. ^*^ *p* < 0.05, ^**^ *p* < 0.01, ^#^ *p* < 0.05, ^##^ *p* < 0.01, data are presented as mean ± standard deviation.

## 3 Results and discussion

### 3.1 Nanoparticle preparation and characterization

**FIGURE 2:**
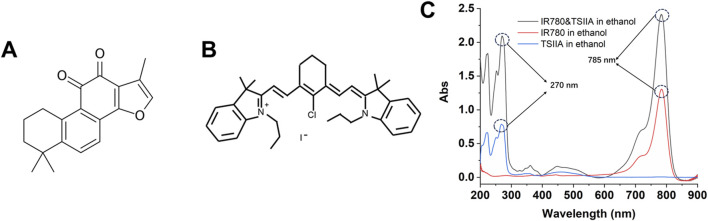
Chemical structures of Tanshinone IIA **(A)** and IR780 **(B)** and their UV characteristic absorption spectra **(C)**.

This experiment employed two model molecules, including the hydrophobic Tanshinone IIA and the near-infrared small molecule IR780. As shown in Figure 2A, Tanshinone IIA belongs to the diterpene class of compounds, featuring a typical diterpene ketone skeleton that includes multiple rings and functional groups. The molecule of Tanshinone IIA contains a ketone group, which is one of the key functional groups contributing to its activity. Tanshinone IIA also features a complex side-chain structure, which may influence its pharmacological activity and bioavailability (Jiang et al., 2019; Huang et al., 2022). The structure of the compound accounts for its multiple biological activities, including anti-inflammatory, antioxidant, antitumor, and antimicrobial properties. IR780 (Figure 2B) is a cyanine dye compound with near-infrared light absorption properties, commonly used in photothermal therapy and biological imaging. The indocyanine group in IR780 molecules is one of the main reasons for its absorption capability in the near-infrared region. Additionally, the presence of an amino group endows IR780 with a certain positive charge, offering unique advantages during the nanoparticle assembly process (Zhang et al., 2014). Figure 2C shows the ultraviolet characteristic absorption peak of Tanshinone IIA at 270 nm, while IR780 has a characteristic absorption peak at 785 nm, with no interference between them, which can be utilized to separate them in quantitative experiments using the differences in ultraviolet characteristic absorption.

According to the literature and experimental procedures, we used UV-vis-NIR spectroscopy to determine IR780, and high-performance liquid chromatography (HPLC) for Tanshinone IIA. As shown in Supplementary Figure S1A, there is a very good linear relationship within a certain concentration range (0.0625–2.0 μg/mL) in organic solvents (*R* ^2^ = 1). From Supplementary Figure S1B, it can be observed that Tanshinone IIA is well separated in the HPLC, with the elution time at 5.5 min and a regular peak shape without significant tailing, which can serve as a quantitative method for Tanshinone IIA.

**FIGURE 3:**
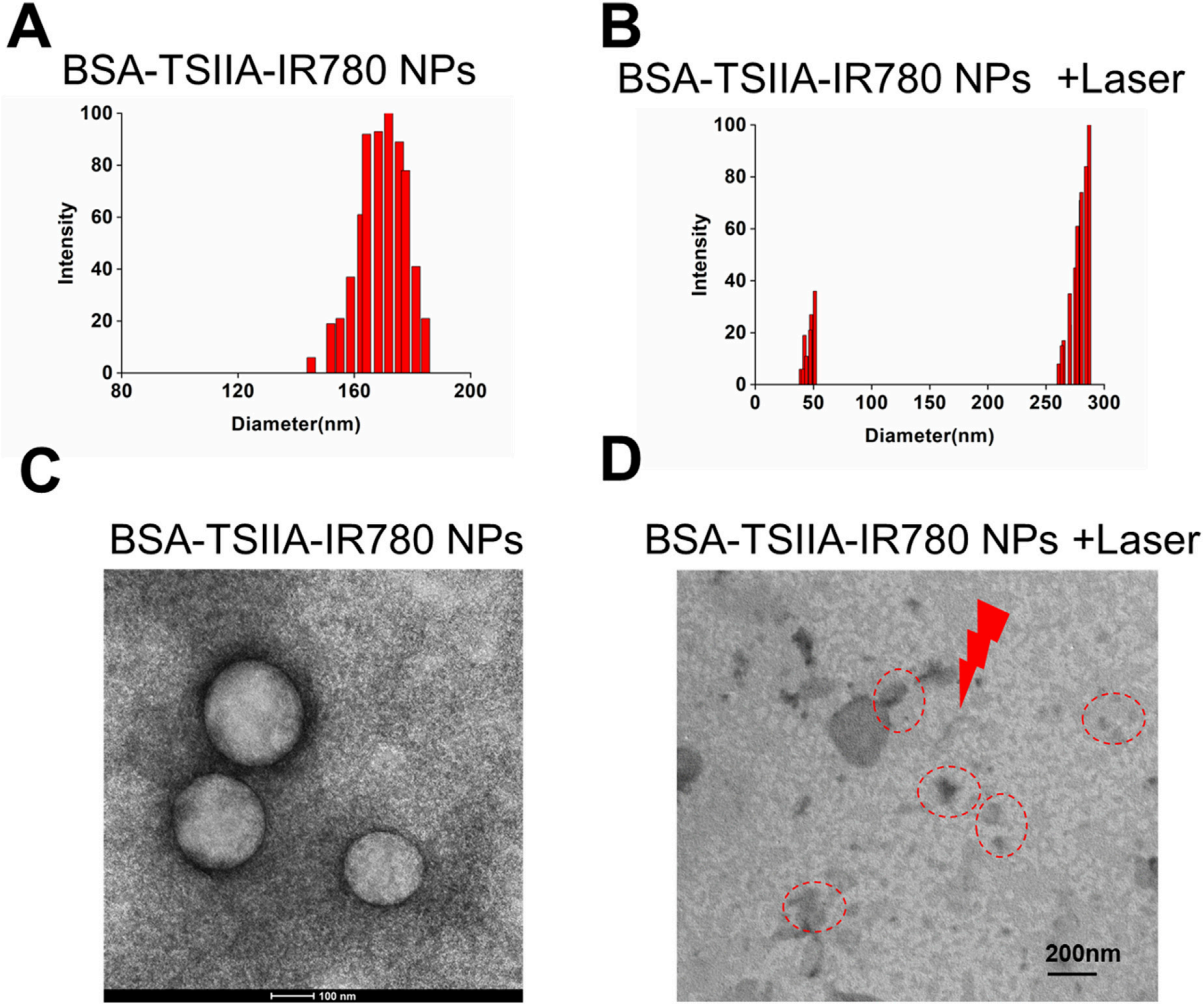
Preparation and characterization of albumin-loaded Tanshinone IIA and IR780 nanoparticles. **(A)**. Particle size and distribution of BSA-TSIIA-IR780 NPs; **(B)**. Particle size distribution of BSA-TSIIA-IR780 NPs after laser irradiation; **(C)**. TEM image of BSA-TSIIA-IR780 NPs; **(D)**. TEM image of BSA-TSIIA-IR780 NPs after laser irradiation.

DTT (dithiothreitol) used in nanoparticle preparation reduces the disulfide bonds in albumin, forming free thiol groups and opening hydrophobic regions. Tanshinone IIA is then added to form initial nanoparticles. IR780, as a hydrophobic charged small molecule, further promotes the assembly of albumin nanoparticles, resulting in drug-loaded nanoparticles with an average diameter of about 185 nm (Figure 3A). Under laser irradiation, IR780 degrades, leading to the “disassembly” of the nanoparticles, which enables the release of Tanshinone IIA. From Figure 3B, it can also be seen that the nanoparticle distribution is broad, with smaller nanoparticles (42 nm) and larger nanoparticles (285 nm), indicating significant changes in the nanoparticles under laser irradiation. The process of IR780 acting as a photosensitizer to generate heat under laser exposure can be explained by the photothermal conversion mechanism. In the presence of a photosensitizer, the laser energy is absorbed and excites the electrons of the photosensitizer to an excited state, forming excited-state photosensitizer molecules. These excited-state molecules, possessing higher energy, release some energy during non-radiative decay, a process known as internal conversion. The released energy is then absorbed by the internal vibrations and rotations of the molecules, causing an increase in internal temperature. This leads to the degradation of the nanoparticles from within, reflected in changes in size and in transmission electron microscopy images (Figures 3C, D). In Figure 3C transmission electron microscopy image, we observed that the albumin nanoparticles were generally uniform and spherical in structure, contributing to their stability in solution. The nanoparticles demonstrated excellent stability in phosphate-buffered saline (PBS) over a period of 7 days, with minimal aggregation or changes in particle size (Narayanan and El-Sayed, 2005). However, after laser irradiation, this regular structure was disrupted, resulting in the appearance of irregular and larger particles. It was demonstrated that after laser irradiation, the structure of the albumin nanoparticles was altered, making the drug more easily released, thereby achieving enhanced therapeutic effects.

### 3.2 Photothermal properties of the nanoparticles

**FIGURE 4:**
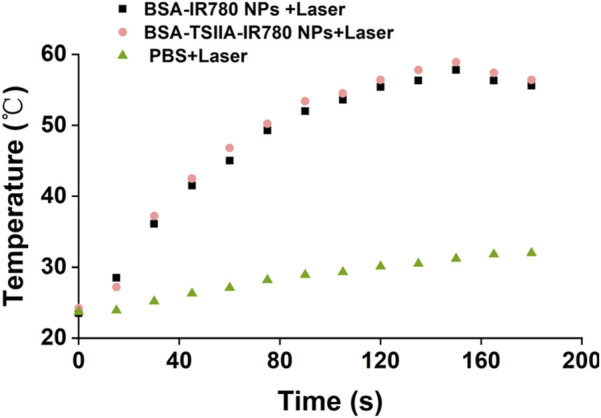
The heating characteristics of BSA-TSIIA-IR780 nanoparticles (0.05 mg/mL IR780), with BSA-IR780 nanoparticles, free IR780, and PBS serving as controls.

In addition to leveraging the anti-inflammatory, antioxidant, antitumor, and antimicrobial biological activities of Tanshinone IIA itself, this project primarily utilizes IR780 as a near-infrared molecule for its heat-producing characteristics. Through photothermal action, it aims to kill bacteria, thus accelerating the wound healing process. Photothermal therapy uses the heat effect produced after a photosensitizer absorbs light to destroy the structure and function of bacterial cells, achieving an antibacterial purpose. Bacteria are sensitive to high temperatures; localized heat effects can cause disruption of bacterial cell membranes, denaturation of proteins, and DNA damage (Xu et al., 2019). Furthermore, high temperatures can also impact bacterial growth and metabolic processes, thereby effectively killing bacteria. Therefore, we evaluated the heat production characteristics of BSA-TSIIA-IR780 NPs in solution (Figure 4). Under conditions of 50 μg/mL IR780 concentration, within 30 s of laser irradiation, the temperature of the BSA-TSIIA-IR780 NPs solution increased from 24°C to 37°C, reached 46.8°C at 60 s, and a maximum of 58.9°C, indicating that under laser irradiation, the photosensitizer IR780 can rapidly heat up and thus kill bacteria to achieve therapeutic purposes.

### 3.3 *In Vitro* release of nanoparticles

**FIGURE 5:**
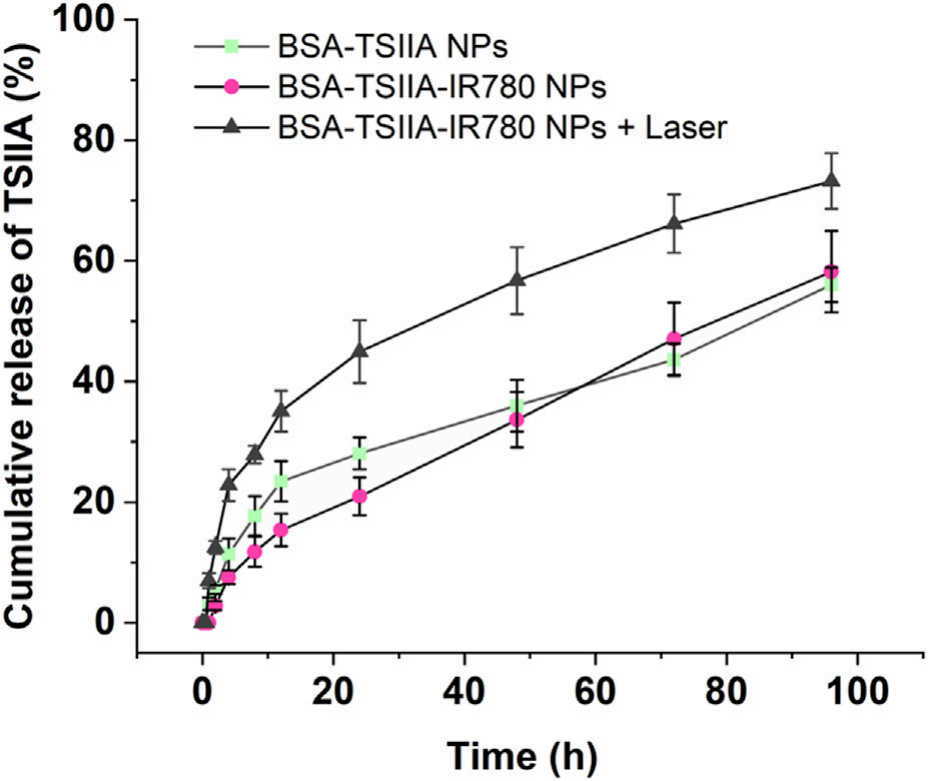
The release study of Tanshinone IIA from nanoparticles with or without laser irradiation. Release medium: PBS containing 1% Tween 80, pH 7.4.

Albumin-loaded nanoparticles containing Tanshinone IIA and IR780 exhibit the capability of enhancing drug release under laser irradiation. Effective release of the drug from the formulation is a prerequisite for its action, and stimulus-responsive release nanopreparations possess the function of “on-demand” and “microenvironment-responsive” release. Therefore, we compared the release behaviors of albumin-loaded Tanshinone IIA nanoparticles and albumin-loaded Tanshinone IIA with IR780 nanoparticles, and verified the laser-responsive release characteristics of the latter (Figure 5). As shown in the figure, compared to the composite nanoparticles, albumin-loaded Tanshinone IIA nanoparticles released Tanshinone IIA faster within 24 h (28.1% vs. 20.9% at the 24th hour), and the trend was similar at the 12th hour (23.7% vs. 15.4%). This may be due to the addition of the small molecule IR780 in the composite nanoparticles, which as a cationic hydrophobic molecule, provides additional forces (such as electrostatic adsorption and hydrophobic interactions) during nanoparticle formation, making it more difficult for Tanshinone IIA to be released from the composite nanoparticles. However, after 48 h, there was no significant difference in the release from the two types of nanopreparations, indicating that the state of nanoparticles in the release medium became consistent after 48 h, as did the behavior of drug release. Under laser irradiation, albumin-loaded nanoparticles containing Tanshinone IIA and IR780 showed a significant increase in release, starting from the first hour after irradiation (12.5% at the second h vs. 2.8%; 35.1% at the 12th hour vs. 15.4%; 56.7% at the 48th hour vs. 33.7%). These results demonstrate that albumin-loaded nanoparticles containing Tanshinone IIA and IR780 have excellent laser-responsive release functionality. This experimental section utilizes albumin to deliver two molecules, IR780 and Tanshinone IIA, leveraging both the heat-producing effect of IR780 under laser irradiation and the antimicrobial and anti-inflammatory pharmacological effects of Tanshinone IIA to treat challenging-to-heal wounds. In the early stages of skin damage, due to potential bacterial infections, there is a need to completely eradicate bacteria and other pathogens, thus photothermal action can meet this requirement; simultaneously, the composite nanoparticles release more Tanshinone IIA under laser irradiation, which has excellent antimicrobial and anti-inflammatory pharmacological effects, allowing better penetration into the skin wound, exerting its effects and regulating the immune microenvironment at the wound site to promote rapid healing and recovery (Huang et al., 2022; Xu et al., 2019).

### 3.4 *In Vitro* antibacterial experiment

**FIGURE 6:**
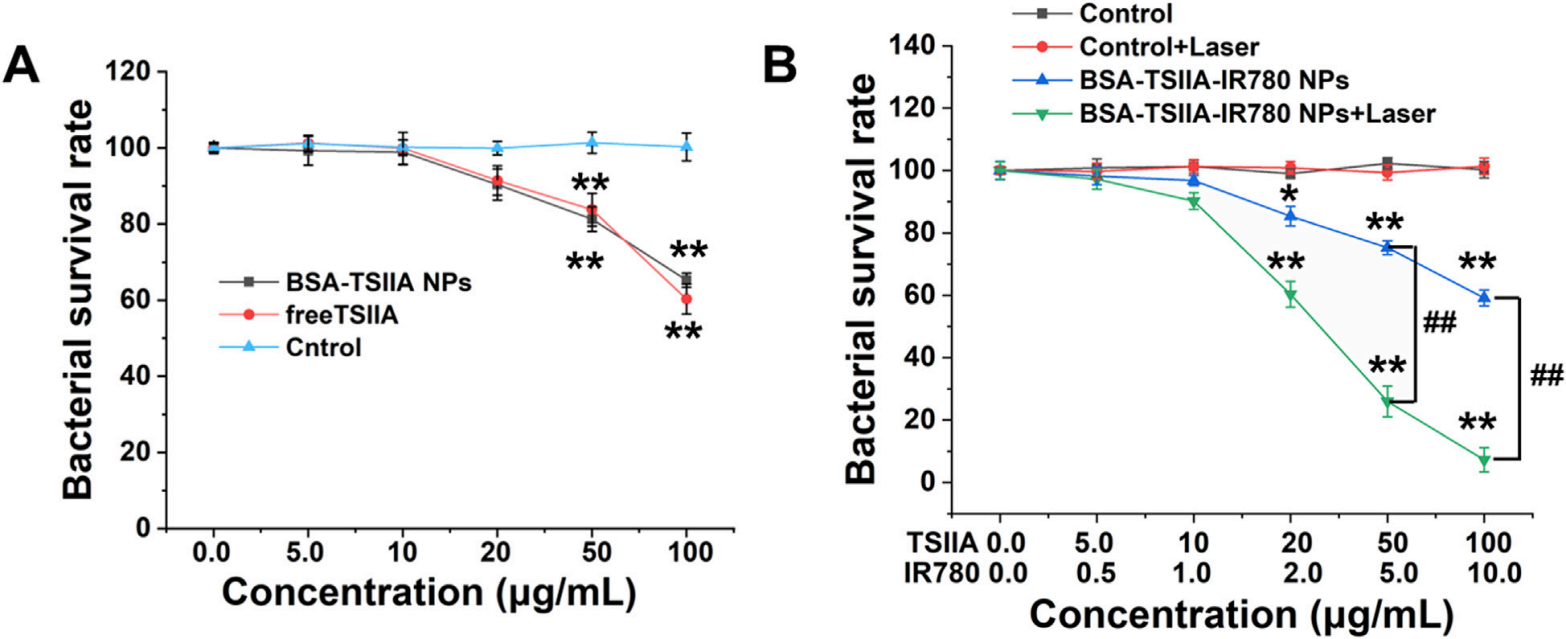
*In vitro* antibacterial characteristics of albumin-loaded Tanshinone IIA and IR780 nanoparticles. **(A)**. Bacterial survival rates at different concentrations of albumin-loaded Tanshinone IIA and free Tanshinone IIA; **(B)**. Bacterial survival rates of albumin-loaded Tanshinone IIA and IR780 nanoparticles with and without laser irradiation. ^*^ *p* < 0.05, ^**^ *p* < 0.01 (vs. control), ^#^ *p* < 0.05, ^##^ *p* < 0.01 (vs. indicated groups).

The *in vitro* antibacterial experiment utilized the CCK-8 assay for measurement. Firstly, we validated the antibacterial properties of Tanshinone IIA. As shown in Figure 6A, both albumin-loaded Tanshinone IIA and free Tanshinone IIA exhibited certain antibacterial effects at high concentrations. At a concentration of 50 μg/mL, the inhibition rate was around 20%, and at 100 μg/mL, the inhibition rate reached 35%–40%. Although there were some antibacterial effects, they were not ideal, hence the addition of photothermal therapy was necessary to further enhance bactericidal activity. From Figure 6B, we observed that the bactericidal capability of the nanoparticles was greatly enhanced with the addition of laser treatment. The laser irradiation group showed significant antibacterial ability even at a Tanshinone IIA concentration of 20 μg/mL (inhibition rate of 40%), and at 100 μg/mL, the inhibition rate reached 93%, indicating that the photothermal effect significantly enhances the antibacterial capability of the nanopreparations. As shown in Figures 3D, 5, the albumin nanoparticles under laser irradiation can more effectively release Tanshinone IIA, which plays an important role in utilizing the antibacterial effects of Tanshinone IIA itself. This is also the main reason why the nanoparticles can achieve a synergistic antibacterial effect under laser irradiation. Upon near-infrared laser irradiation, the IR780 generates localized heat, increasing the temperature of the bacterial environment. The heat generated causes damage to the bacterial cell membrane by increasing membrane fluidity and permeability, ultimately leading to cell lysis. The loss of membrane integrity compromises the bacteria’s ability to maintain homeostasis, which is critical for survival (Xu et al., 2023). This experiment demonstrates that the antibacterial performance of albumin-loaded nanoparticles containing Tanshinone IIA and IR780 primarily relies on the heat generation capability of the near-infrared small molecules, while Tanshinone IIA mainly plays a role in anti-inflammatory responses, scavenging free radicals, and regulating immune therapeutic effects in treatment. The nanoparticles enhance bacterial clearance through the synergistic action of Tanshinone IIA’s antimicrobial properties and the photothermal effect of IR780. Under near-infrared laser irradiation, IR780 generates localized heat that leads to bacterial cell membrane disruption and protein denaturation, while Tanshinone IIA further inhibits bacterial growth (Xu et al., 2023).

### 3.5 *In Vivo* wound healing experiment in mice

**FIGURE 7:**
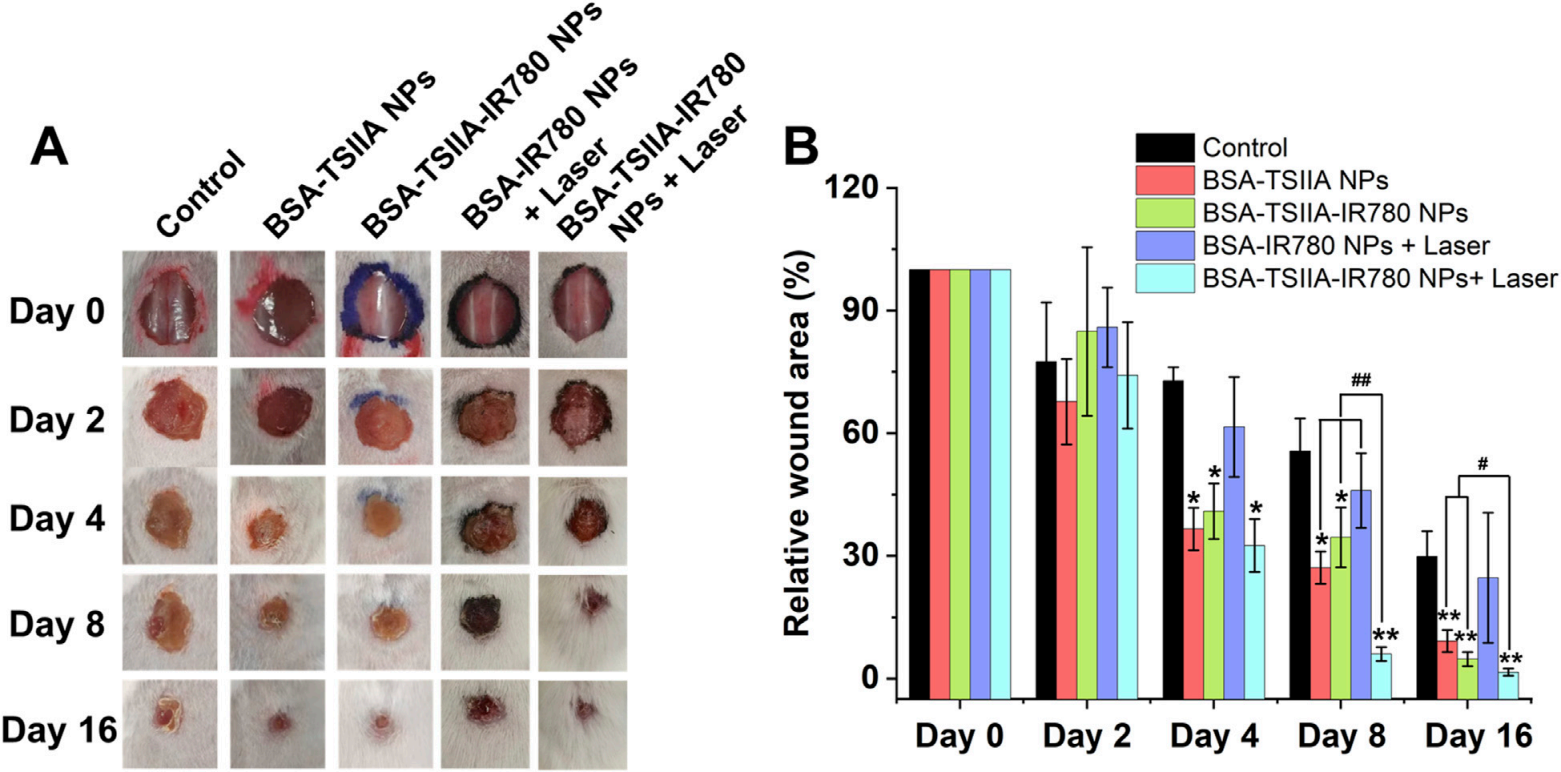
Therapeutic effects of albumin-loaded Tanshinone IIA and IR780 nanoparticles in a mouse wound healing model infected with MRSA bacteria. **(A)**. Representative images of the wound healing process under different treatment regimens; **(B)**. Relative wound areas for each group at different time points. ^*^ *p* < 0.05, ^**^ *p* < 0.01 (vs. control), ^#^ *p* < 0.05, ^##^ *p* < 0.01 (vs. indicated groups).

We validated the combined effects of nanoparticle-based photothermal therapy and the antibacterial properties of Tanshinone IIA on enhancing wound healing in a mouse wound healing model. The MRSA infected mouse wound model is characterized by its difficulty in healing (Figure 7A Control group), and by the 16th day after establishing the wound model, the relative wound area was 29.9%, confirming the successful establishment of this model. Our treatment group, with albumin-loaded Tanshinone IIA nanoparticles, showed a significantly better therapeutic effect, with a relative wound area of 9.2% on the 16th day, demonstrating the nanoparticles’ excellent capability to promote wound healing and the important role of Tanshinone IIA in wound healing (Figure 7B). In the group treated with albumin-loaded Tanshinone IIA and IR780 nanoparticles, we obtained similar results to the Tanshinone IIA nanoparticle group, with a relative wound area of 4.8% on the 16th day, and statistical results showed no significant difference from the Tanshinone IIA nanoparticle group. In the laser irradiation group, we found that the albumin IR780 nanoparticles without Tanshinone IIA performed no differently from the Control group, proving that photothermal therapy alone under this study’s conditions does not significantly enhance wound healing in mice, with a relative wound area of only 24.7% on the 16th day. However, the final treatment group, albumin-loaded Tanshinone IIA and IR780 nanoparticles combined with laser treatment, leveraging the dual advantages of photothermal sterilization and enhanced release of Tanshinone IIA, exhibited significant antibacterial performance and greatly enhanced the healing of difficult wounds (16th day, relative wound area of only 1.5%). Beyond bacterial clearance, Tanshinone IIA is known for its anti-inflammatory properties. It helps regulate the inflammatory response by reducing the infiltration of inflammatory cells and downregulating pro-inflammatory cytokines, which prevents chronic inflammation and promotes the transition to the proliferation phase of wound healing. This anti-inflammatory effect accelerates tissue repair by reducing tissue damage caused by excessive inflammation (Guo et al., 2020). The results prove that our designed composite nanoparticles have an excellent ability to promote wound healing.

## 4 Conclusion

We have reported a nanoparticle system composed of albumin loaded with Tanshinone IIA, a monomeric active molecule derived from traditional Chinese medicine, and IR780, a near-infrared small molecule capable of producing photothermal effects. These nanoparticles were prepared using molecular self-assembly and applied to bacterial-infected wound healing. Albumin-loaded Tanshinone IIA and IR780 nanoparticles demonstrated good photothermal properties, biocompatibility, and excellent antibacterial activity under near-infrared irradiation, suggesting potential clinical applications in wound dressings. Various characterization methods, including electron microscopy, particle size distribution, and UV spectroscopy, confirmed the successful creation of these functionalized nanoparticles. Compared to the absence of laser irradiation, the albumin-loaded Tanshinone IIA and IR780 nanoparticles enhanced the release of Tanshinone IIA under near-infrared light, indicating that this nanoparticle combination possesses near-infrared light-activated drug release properties. The photothermal effect, along with the antibacterial, anti-inflammatory, and free radical scavenging activities of Tanshinone IIA, which also modulate the immune microenvironment of the wound, enhanced the antibacterial behavior both *in vitro* and *in vivo*. Overall, these results suggest that protein-loaded Tanshinone IIA and IR780 nanoparticles under laser irradiation may be an ideal therapeutic approach for enhancing the healing of bacterial-infected wounds.

Tanshinone IIA is an effective traditional Chinese medicine extract with multiple biological activities. Advancing its clinical application is a responsibility for every researcher and clinician; however, its poor water solubility requires the use of organic solvents for dissolution, which can lead to potential toxic side effects. Albumin has multiple binding sites for hydrophobic drugs, naturally making it a carrier for hydrophobic drug delivery. Albumin nanoparticle formulations have also gained widespread clinical use, such as commercially available albumin-bound paclitaxel nanoparticles and albumin-bound rapamycin nanoparticles (Fu et al., 2022). Therefore, developing an albumin-based nanoparticle formulation holds significant clinical value. This part of the study started from effective components of traditional Chinese medicine, combined with the advantages of nanoparticle formulations, and applied the relatively novel photothermal therapy to develop albumin-loaded Tanshinone IIA composite nanoparticles, which were comprehensively evaluated and preliminarily validated *in vivo*. In addition to its antimicrobial and anti-inflammatory actions, Tanshinone IIA has been shown to promote angiogenesis. By stimulating endothelial cell proliferation and migration, Tanshinone IIA contributes to the formation of new blood vessels, enhancing oxygen and nutrient supply to the wound site, which is critical for tissue regeneration and accelerated wound healing (Guo et al., 2020). However, further improvements are needed for this project, such as explaining the mechanisms by which Tanshinone IIA promotes wound healing and how photothermal therapy synergistically enhances the pharmacological actions of Tanshinone IIA. These aspects will be further explored in our subsequent work. While the results are promising, translating this nanoparticle system into clinical practice poses several challenges. These include scaling up production, ensuring consistent drug release profiles, and conducting rigorous clinical trials to verify safety and efficacy in human patients. Overcoming these challenges will be essential for the potential clinical application of this system in treating chronic and infected wounds.

## Data Availability

The original contributions presented in the study are included in the article/Supplementary Material, further inquiries can be directed to the corresponding authors.
