# Supplementary material for: Photothermally enhanced antibacterial wound healing using albumin-loaded tanshinone IIA and IR780 nanoparticles
Source: Front Bioeng Biotechnol. 2024 Oct 23;12:1487660. doi: 10.3389/fbioe.2024.1487660 (PMC11538050; doi:10.3389/fbioe.2024.1487660)
Supplement: Supplementary file 1 [file DataSheet1.docx]

**Photothermally Enhanced Antibacterial Wound Healing Using Albumin-Loaded Tanshinone IIA and IR780 Nanoparticles**

Haidong Chen^1^, Yimei Li^1^, Dexuan Chen^2^, Yong Fang^1^, Xuchu Gong^1^, Kaikai Wang^1,3*^, Chaoqun Ma^2*^


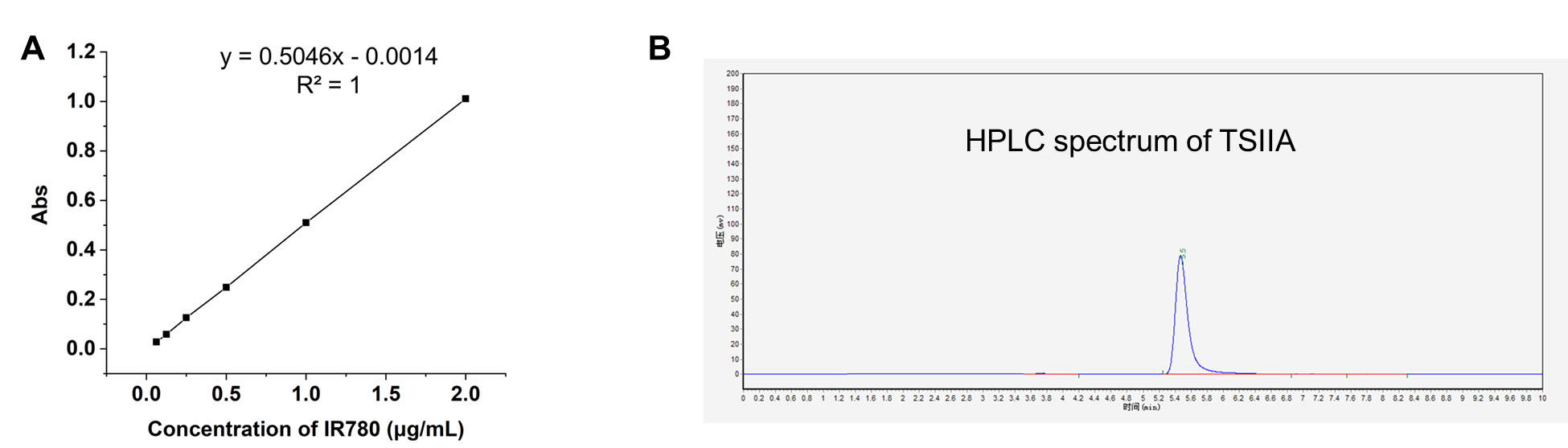


Figure S1. A. UV standard curve of IR780 in organic solvent; B. HPLC chromatogram of Tanshinone IIA.
